# Old for young kidney transplantation: a responsible option for our patients to reduce waiting time?

**DOI:** 10.1007/s00345-024-04779-8

**Published:** 2024-02-16

**Authors:** Philip Zeuschner, Janine Mihm, Urban Sester, Michael Stöckle, Frank Friedersdorff, Klemens Budde, Abdulbaki Yakac, Christian Thomas, Johannes Huber, Juliane Putz, Luka Flegar

**Affiliations:** 1 Department of Urology and Pediatric Urology, Saarland University, Kirrberger Street 100, 66421 Homburg/Saar, Germany; 2 Medical Department III: Renal and Hypertensive Diseases, Immunology and Dialysis, SHG Kliniken Völklingen, Richardstraße 5-9, 66333 Völklingen, Germany; 3 Department of Urology, Charité-Universitätsmedizin Berlin, Corporate Member of Freie Universität Berlin, Humboldt-Universität zu Berlin, and Berlin Institute of Health, Charitéplatz 1, 10117 Berlin, Germany; 4 Department of Nephrology, Charité-Universitätsmedizin Berlin, Corporate Member of Freie Universität Berlin, Humboldt-Universität zu Berlin, and Berlin Institute of Health, Charitéplatz 1, 10117 Berlin, Germany; 5 Department of Urology, University Hospital Carl Gustav Carus, Technische Universität Dresden, Fetscherstraße 74, 01307 Dresden, Germany; 6 Department of Urology, Philipps University of Marburg, Baldingerstraße, 35043 Marburg, Germany

**Keywords:** Dialysis, Kidney transplantation, Renal insufficiency, Old for young kidney transplantation, Eurotransplant senior program

## Abstract

**Purpose:**

The Eurotransplant Senior program allocating grafts from donors ≥ 65 years to recipients aged ≥ 65 years has proven good results within the last 20 years. However, “old” grafts are also allocated to younger recipients < 65 years, and this outcome of “old for young” kidney transplantations (KT) still lacks detailed investigations.

**Methods:**

All “old for young” KT performed at four tertiary referral centers were retrospectively compared including a recent follow-up, stratifying for “old for young” (donor ≥ 65 years to recipient < 65 years) vs. “very old for young” KT (donor ≥ 70 years to recipient < 65 years).

**Results:**

Overall, 99 patients were included with 56 (56.6%) “old for young” and 43 (43.4%) “very old for young” KT. The median waiting time did not differ (60.7 vs. 45.8 months, respectively) at comparable living donation rates (57.1% vs. 44.2%) as well as intra- and postoperative results. At a median follow-up of 44 months (range 1; 133), the 3-year graft survival of 91% vs. 87% did not significantly vary. In subgroup analyses assessing living donation or donation after brain death (DBD) KT only, the graft survival was significantly longer for “old for young” KT within the living donation subgroup. In multivariate Cox regression analyses, the presence of panel-reactive antibodies was the only significant impact factor on graft survival (HR 8.32, p = 0.001).

**Conclusion:**

This analysis clearly demonstrates the effectiveness of the "old for young" approach, enabling favorable perioperative results as well as comparable data of graft- and overall survival, while reducing waiting time for eligible patients.

**Supplementary Information:**

The online version contains supplementary material available at 10.1007/s00345-024-04779-8.

## Introduction

Kidney transplantation (KT) is considered the gold standard treatment for patients with end-stage renal disease (ESRD), providing higher survival rates and a better quality of life compared to dialysis [1]. In Germany, the allocation and cross-border exchange of deceased donor organs is organized by Eurotransplant, an international non-profit organization based in the Netherlands [2]. Since 1969, Eurotransplant aims to enhance solid organ transplantation outcomes within an enlarged graft pool of eight European countries. For special patient groups, separate allocation algorithms were established: the Acceptable Mismatch Program (AM) and European Senior Program (ESP) were introduced besides the Eurotransplant Allocation System (ETKAS) in 1996 and 1999 [3–7].

The ESP or “old for old-program, was specifically developed to reduce waiting times for recipients aged ≥ 65 years by exclusively allocating kidneys from deceased donors ≥ 65 years [8]. Since its introduction in 1999, its outcomes were acceptable, even during COVID-pandemic [9, 10]. Each year, about 500 kidneys are transplanted in this program [11]. However, not only the number of dialysis patients aged ≥ 65 years who are potentially eligible for KT and consequently the ESP program, but also the number of kidney donors older than ≥ 65 years disproportionately grew within the last years, especially in Germany. Consequently, some organs can no longer be allocated within ESP due to a lack of recipients, wherefore recipients < 65 years increasingly receive an organ offer. However, the outcome of this “old for young” KT has not been thoroughly investigated [7].

To this end, we retrospectively analyzed all “old for young” kidney transplantations performed at four tertiary referral centers between 2010 and 2022 and compared the mid- and long-term kidney function as well as the perioperative outcomes.

## Materials and methods

All kidney transplantations from donors ≥ 65 years to recipients < 65 years performed at four tertiary referral centers (at Homburg/Saar, Berlin, Dresden, Marburg) were retrospectively included from 2010 to 2022. Both living and deceased donor KT were included, but not double KT. All KT were conducted in an open approach by experienced transplant surgeons. All kidney recipients received basiliximab as induction therapy in combination with tacrolimus, mycophenolate mofetil and glucocorticoids as the standard immunosuppressive regimen at the respective transplant centers.

The recipient (relevant health conditions, cause for ESRD, duration / type of dialysis, number of prior KT), donor and graft characteristics (HLA-mismatches, cold ischemia time (CIT)) were obtained. Besides the surgical outcomes of KT (operating time, warm ischemia time (WIT), intraoperative complications), postoperative data were collected (complications based on Clavien Dindo). The graft function was assessed by delayed graft function rates (DGF; defined as the need for at least two haemodialyses within 7 days after transplantation). A recent follow-up including was collected, the serum creatinine, death-censored graft and patient survival.

As the primary outcome, the mid- and long-term kidney function were assessed and compared between “old for young” (donor ≥ 65 years to recipient < 65 years) and “very old for young” (donor ≥ 70 years to recipient < 65 years) KT. As the secondary outcome, the perioperative results were compared between the groups. Graft and overall survival were further assessed within the subgroups living donation or donation after brain death (DBD) only and the impact of various factors on graft and overall survival was estimated by uni- and multivariate Cox regression analyses.

Categorical variables were reported as frequencies and proportions, continuous data as the median and range. Fisher’s exact test and Mann–Whitney-U-Test were used for pairwise comparisons. Death-censored graft and patient survival were estimated by Kaplan Meier estimates and compared between groups by log-rank tests. All statistical analyses were performed by SPSS version 29 (IBM, Armonk, NY, USA), all tests were two-sided, and p values < 0.05 were considered as significant.

The analysis was conducted in adherence with the scientific research work teams of the respective centers. This study was conducted according to the Declaration of Helsinki and approved by the local responsible ethical review boards. Patient data were fully anonymized.

## Results

### Patient cohort

Overall, 99 patients were included (Homburg: n = 31, Berlin: n = 25, Dresden n = 29, Marburg n = 14,) with 56 (56.6%) “old for young” and 43 (43.4%) “very old for young” KT. When comparing the donor, recipient and graft characteristics of “old” vs. “very old for young” KT, only the age gap between donor and recipient significantly differed between the groups (12.5 vs. 22 years, p < 0.001; Table 1). The living donation rates were comparable between “old” vs. “very old for young” KT (57.1 vs. 44.2%). In both groups, about 15% of KT were performed in a pre-emptive manner as living kidney donation (16.1 vs. 11.6%). The remaining patients had a comparable median waiting time of 60.7 vs. 45.8 months.

**Table 1:** Donor, recipient and graft characteristics, stratified by “old for young” and “very old for young” KT

	Total (n = 99)	“Old for young “ (n = 56)	“Very old for young” (n = 43)	p value
Donor age [yr]	69 (65; 84)	67 (65; 69)	73 (70; 84)	** < 0.001**
Recipient age [yr]	54 (13; 64)	55 (25; 64)	54 (13; 64)	0.452
Age gap donor-recipient	20 (1; 63)	12.5 (1; 40)	22 (9; 63)	** < 0.001**
Male recipient	60 (60.6%)	35 (62.5%)	25 (58.1%)	0.683
BMI [kg/m^2^]	25.2 (17.1; 69)	25.9 (17.5; 69)	25 (17.1; 36.9)	0.251
Underlying disease				0.619
Chronic GN	18 (18.2%)	10 (17.9%)	8 (18.6%)	0.563
IgA nephropathy	12 (12.1%)	6 (10.7%)	6 (14%)	0.425
ADPKD	10 (10.1%)	4 (7.1%)	6 (14%)	0.323
Other	15 (15.2%)	10 (17.9%)	5 (11.6%)	0.573
Hypertension	84 (84.8%)	49 (87.5%)	35 (81.4%)	0.287
CAD	14 (14.1%)	6 (10.7%)	8 (18.6%)	0.204
Diabetes	8 (8.1%)	5 (8.9%)	3 (7%)	0.513
History of smoking	17 (17.2%)	9 (16.1%)	5 (11.6%)	0.472
≥ 1 prior KT	9 (9.1%)	4 (7.1%)	5 (11.6%)	0.336
Waiting time [mo]	51.4 (0.23; 255)	60.7 (1.4; 255.5)	45.8 (0.23; 130.1)	0.421
Type of dialysis				0.347
Haemodialysis	69 (69.7%)	38 (67.9%)	31 (72.1%)	
CAPD	4 (4%)	1 (1.8%)	3 (5%)	
Peritoneal dialysis	12 (12.1%)	8 (14.3%)	4 (9.3%)	
Living donation	51 (51.5%)	32 (57.1%)	19 (44.2%)	0.141
Pre-emptive KT	14 (14.1%)	9 (16.1%)	5 (11.6%)	0.371
Sum of HLA-MM	3 (0; 6)	3 (0; 6)	3 (0; 6)	0.496
Presence of PRA	17 (17.2%)	8 (14.3%)	9 (20.9%)	0.287

*BMI* body mass index, *HLA-MM* number of Human leukocyte antigen mismatches, *PRA* panel-reactive antibodies, *CAD* coronary artery disease, *CAPD* continuous ambulatory peritoneal dialysis, *KT* kidney transplantation

### Intra- and postoperative results

As for the intra- and postoperative results, the “old” and “very old for young” groups did not significantly differ (Table S1). Intraoperative complications occurred in 9% of cases (Table S2), postoperative complications in 37.5% vs. 44.2%, respectively. Clavien Dindo grade 2 complications were most frequent (21.4 vs. 27.9%). Major complications were rare, including one fascia dehiscence, one urinary retention, two lymphocele resections, one arterial re-anastomosis on postoperative day 1, one thromboendarterectomy of the transplant artery directly postoperative, one arteriovenous fistula on postoperative day 20 after transplant biopsy treated with coiling, one reflux esophagitis and one postoperative hematoma with surgical re-exploration on the first postoperative day. One patient had DGF with prolonged haemodialysis; but the DGF rates did not differ between the groups (35.7% vs. 37.2%; Table S1).

### Follow-up

**Fig. 1:**
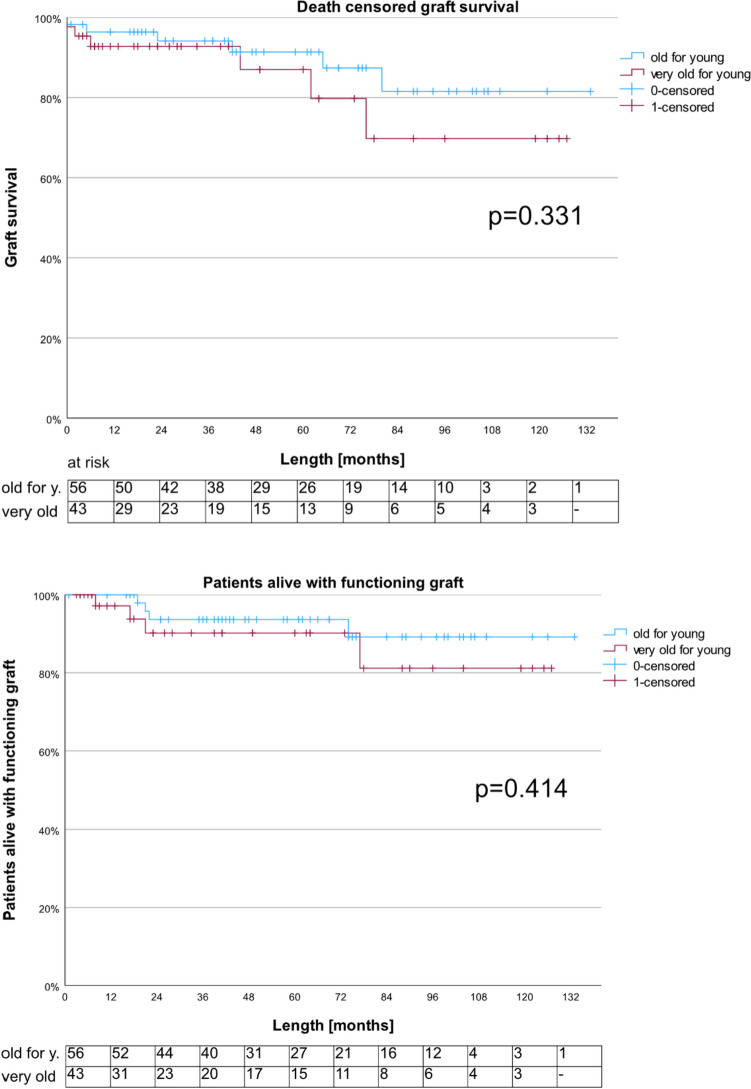
Death-censored graft and patient survival, compared between “old for young” vs. “very old for young” KT; the respective number of patients at risk is given below each graph

At a median follow-up of 44 months (range 1; 133), there were 12 (12.1%) graft losses in both groups, six each. At a 3-year graft survival of 91.4% (95% CI 78.2; 96.7) vs. 87% (66; 95.4), it did not significantly differ between “old” vs. “very old for young” KT (Fig. 1, Table S3).

Correspondingly, levels of the initial creatinine were comparable before KT [“old for young”: median 7.3 mg/dl (range 4; 18.3) vs. “very old for young”: 6.8 mg/dl (range 3.4; 14.1)], but also at one [1.6 mg/dl (0.8; 10.8) vs. 1.8 (1.0; 10.6)], three [1.8 mg/dl (0.7; 3.4) vs. 1.8 (1.2; 11.7)] and five years post KT [1.7 mg/dl (1.2; 4.2) vs. 1.5 (1.1; 3.4); Fig. S1].

Neither did the patient survival differ between the two groups, it was 93.7% (95% CI 81.6; 97.9) vs. 90.2% (72.3; 96.8) after three and 89.2% (71.9; 96.1) vs. 81.2% (52.1; 93.5) after five years (Fig. 1, Table S3).

### Subgroup analysis: living kidney donation vs. donation after brain death

When comparing living donation vs. DBD KT, the recipient age was significantly lower for living KT (44 vs. 58.5 years, p < 0.001) and the age gap donor-recipient was significantly greater (25 vs. 11 years, p < 0.001, Table S3). The proportion of “old” vs. “very old for young” KT did not significantly differ. Patients undergoing living KT had a lower BMI, coronary artery disease less often and a shorter waiting time (Table S4). As for the surgical results, there was a shorter cold ischemia (152.5 vs. 906 min, p < 0.001) and operating time (154 vs. 189 min, p < 0.001; Table S5) during living KT. Complications were more frequent after DBD, especially Grade 3a complications (0 vs. 10.4%, p = 0.024). The DGF rates were almost three times higher after DBD KT (19.6 vs. 54.2%, p < 0.001).

When comparing graft and patient survival between “old” vs. “very old for young” KT within the subgroups DBD or living kidney donation, the graft survival was only significantly longer for “old for young” KT within the living donation subgroup (Fig. S2). Here, the 3-year graft survival was 100% vs. 100% at 3 years, but 100% vs. 84.6% at 5 years (p = 0.020, Table S6). Neither the patient survival within the living donation subgroup nor the survival in the DBD subgroup significantly differed (Fig. S2, Table S6).

### Influencing factors on graft and patient survival

In multivariate Cox regression, only the presence of panel-reactive antibodies had a significant impact on graft survival (HR 8.32, p = 0.001, Table S7). The waiting time (HR 1.16, p = 0.018) and a preexisting coronary artery disease (HR 5.47, p = 0.004) also increased the risk for early graft loss, but in univariate Cox regression only. In contrast, neither the type of kidney donation (DBD vs. living) nor the donor or recipient age had a significant impact.

For the patient survival, an increasing BMI lowered the risk for patient death (HR 0.73, p = 0.008). In contrast, it was significantly shortened by a pre-existing coronary artery disease (HR 19.74, p < 0.001) in multivariate Cox regression analysis (Table S8). The waiting time (HR 1.17, p = 0.027) and presence of PRA (HR 4.44; p = 0.035) only exerted a significant on the patient survival impact in univariate Cox regression analysis.

## Discussion

The Eurotransplant Senior Program was introduced in 1999 to improve the probability of an organ offer for kidney recipients aged ≥ 65 years. Almost 25 years later, the acceptance of organ donors ≥ 65 years has considerably enlarged the donor pool and reduced the waiting time to an average of 50 months, whereas younger patients in ETKAS must wait an average of 98 months for KT in some German transplant centers. However, if there is no suitable recipient in ESP, the grafts are also offered to patients < 65 years, which raises the question of the outcomes of this “old for young” kidney transplantation. In the present evaluation, we investigated the outcomes of 99 such “old for young” KT at four kidney transplant centers. To assess the impact of the donor age, we further defined two distinct groups comparing “old for young” (donor ≥ 65 years to recipient < 65 years) and “very old for young” (donor ≥ 70 years to recipient < 65 years) KT. In brief, the results were favorable with good perioperative outcomes, and the graft and overall survival were comparable between the two subgroups.

First of all, the 3-year graft (91% vs. 87%) and patient survival (94% vs. 90%) proved good results both for the “old” and “very old for young” KT. These results also remained stable five years post KT compared to published data [12]. Within the last decades, the typical kidney donor has considerably aged [13, 14]. For living kidney donation, the acceptance of such older, but otherwise healthy donors is nowadays well accepted [15, 16]. This is also the case in our population with high living kidney donation rates not only within the “old” (53.6%), but also the “very old for young” subgroup (35.7%). However, data from deceased kidney donation of older donors to younger recipients are still lacking.

Hence, we further conducted respective subgroup analyses to assess the impact of the type of kidney donation, namely DBD vs. living kidney donation. Here, the graft survival only significantly differed between “old” vs. “very old for young” KT in the living donation subgroup. In contrast, neither the patient nor graft survival differed within the subgroup of DBD KT only. Ultimately, it appears to be advantageous for all recipients to have an “old” or at least “very old” donor, as the graft survival was at least 78.9% after 5 years for “old for young” within DBD. According to CTS data, the 5-year graft survival is about 76.1% for deceased donors in general [17]. Consequently, our data illustrates a comparable or even slightly superior outcome even if the age gap was considerably high between “very old to young” donors in some subgroups.

Second, there were no significant differences in intra- and postoperative results between the “old” and “very old for young” groups. Intraoperative complications were observed in 9% of cases, while postoperative complications occurred in 37.5% and 44.2%, respectively. Overall, peri- and postoperative major complications were rare, primarily dependent on the recipient’s age rather than the donor’s age. Of note, none of the complications were directly associated with donor age.

As DGF is a matter of age as shown by Kernig et al., we expected DGF rates to be higher in the “very old for young” group [18]. However, the DGF rates were not significantly higher when comparing “old” vs. “very old for” KT (DGF rate 35.7% vs. 37.2%, respectively). Besides, DGF itself is mostly driven by ischemic-reperfusion injury as well as immunological factors [19, 20]. Allocation of older kidneys to younger patients with good HLA compatibility may reduce these factors at the benefit of a much shorter waiting time as the third aspect of this evaluation.

Waiting time is a well-known major factor reducing graft and patient survival. In our analysis, the waiting time was longer for “old for young” KT compared to “very old for young” KT (60.7 vs. 45.8 months), but yet not statistically different. Of note, the median waiting time for a typical transplant recipient nowadays ranges between 8 and 10 years in Germany—which clearly emphasizes, that old for young KT in Germany may reduce waiting times by enlarging the donor pool. This further exerts an important side effect, as an earlier kidney transplantation may stop the progression of coronary artery disease by reducing the end-organ damage caused by dialysis [21]. Correspondingly, coronary artery disease exerted a massive impact on patient survival in this analysis. However, the waiting time itself did not remain a statistically significant impact factor in the multivariate analysis. Of note, the main focus of ESP was to shorten the cold ischemia times by local allocation, against the tradeoff of not to consider the HLA status. This clearly led to the benefit of shorter waiting times, but on the other hand it deteriorated the immunological compatibility of the grafts. Our analysis showed no differences in the HLA mismatches between the groups, so the only significant impact on graft loss was measurable for PRA status. Correspondingly, the presence of PRA had the strongest impact on graft loss in multivariate Cox regression analysis. This is a well-known aspect in the literature [22]. Hence, according to our findings, the presence of PRA could be one of the decision criteria when considering an “old for young” transplantation for our patients.

While the current data provides valuable observations regarding old for young KT and underlines its rationale, it is essential to acknowledge its inherent limitations and to interpret the findings within the context of its retrospective, multi-institutional design. First, the patient number was limited and second, although the patient cohort was well characterized, there was missing data about the donor characteristics, such as cause of death or comorbidities.

To conclude, our data clearly demonstrate that “old for young” kidney transplantation does not only represent a feasible, but also reasonable approach to reduce waiting times for potential kidney recipients. It may further enable favorable perioperative results as well as comparable data of graft- and overall survival also for “very old to young” kidney transplantation. The graft and patient survival ultimately appear to be almost comparable to kidney transplants among younger donors and recipients. However, one must keep in mind that a wise donor selection remains a significant prerequisite in this context. Furthermore, it is major aspect to know the own patients on the waiting list particularly well in order to enable a favorable patient selection. Nevertheless, against the background of these potential advantages of an “old for young” constellation, such organ offers should always be seen, albeit critically, as a great opportunity for our patients.

## Supplementary Information

Below is the link to the electronic supplementary material.

Supplementary file1 (DOCX 858 KB)

## Data Availability

Data is availiable upon reasonable request.

## Abbreviations

BMI: Body mass index
CIT: Cold ischemia time
CAPD: Continuous ambulatory peritoneal dialysis
DBD: Donation after brain death
DGF: Delayed graft function
ESP: Eurotransplant Senior Program
ESRD: End-stage renal disease
HLA-MM: Number of human leukocyte antigen mismatches
KT: Kidney transplantation
PRA: Panel-reactive antibodies
WIT: Warm ischemia time
