# Supplementary material for: Old for young kidney transplantation: a responsible option for our patients to reduce waiting time?
Source: World J Urol. 2024 Feb 16;42(1):85. doi: 10.1007/s00345-024-04779-8 (PMC10873431; doi:10.1007/s00345-024-04779-8)
Supplement: Supplementary file 1 — Supplementary file1 (DOCX 858 KB) [file 345_2024_4779_MOESM1_ESM.docx]

# Supplementary Material

## Table S1. Intra- and postoperative results, stratified by “old for young” vs. “very old for young” subgroup.

|  | **Total (n=99)** | **“Old for young“ (n=56)** | **“Very old for young” (n=43)** | **p-value** |
| --- | --- | --- | --- | --- |
| left-sided implantation | 36 (36.4%) | 22 (39.3%) | 14 (32.6%) | 0.620 |
| CIT [min] | 233 (4; 1783) | 207 (72; 1783) | 483 (4; 1440) | 0.740 |
| operating time [min] | 171 (84; 472) | 165.6 (84; 267) | 181 (11; 472) | 0.331 |
| WIT [min] | 38.5 (17; 105) | 37 (19; 98) | 39 (17; 105) | 0.489 |
| intraoperative complications | 9 (9.1%) | 5 (8.9%) | 4 (9.3%) | 0.608 |
| postoperative complications classified as Clavien Dindo | 40 (40.4%) | 21 (37.5%) | 19 (44.2%) | 0.368 |
| grade 1 | 6 (6.1%) | 4 (7.1%) | 2 (4.7%) | 0.472 |
| grade 2 | 24 (24.2%) | 12 (21.4%) | 12 (27.9%) | 0.304 |
| grade 3a | 5 (5.1%) | 3 (5.4%) | 2 (4.7%) | 0.624 |
| grade 3b | 5 (5.1%) | 3 (5.4%) | 2 (4.7%) | 0.624 |
| ICU | 35 (35.4%) | 21 (37.5%) | 14 (32.6%) | 0.417 |
| length ICU stay [d] | 5 (0; 29) | 5 (0; 29) | 5 (0; 15) | 0.413 |
| DGF | 36 (36.4%) | 20 (35.7%) | 16 (37.2%) | 0.835 |

CIT: cold ischemia time, WIT: warm ischemia time, ICU: intensive care unit, DGF: delayed graft function

Table S2. Intraoperative complications.

|  | Type of intraoperative complication |
| --- | --- |
| “old for young” | - n=3 re-anastomosis of transplant artery due to absence of kidney perfusion due to arteriosclerosis and intima dissection - 2cm cut in renal parenchyma |
| “very old for young” | - n=1 re-anastomosis of transplant artery due to massive arteriosclerosis and intima dissection - Ureteral anastomosis complex - massive arteriosclerosis – long anastomosis time – 2 erythrocyte concentrates - intraoperative bleeding – 4 erythrocyte concentrates |

## Table S3. One, three- and five-years death censored graft survival and overall survival, compared between “old for young” vs. “very old for” young KT, including the 95% confidence intervals.

|  | **“Old for young” KT** | **“Very old for young” KT** |
| --- | --- | --- |
| **Death censored graft survival** |  |  |
| 1 year | 96.4% (86.2; 99.1) | 92.8% (79.2; 97.6) |
| 3 years | 91.4% (78.2; 96.7) | 87% (66; 95.4) |
| 5 years | 87.4% (71; 94.8) | 79.7% (66; 95.4) |
| **Patient survival** |  |  |
| 1 year | 98% (86.1; 99.7) | 97.1% (81.4; 99.6) |
| 3 years | 93.7% (81.6; 97.9) | 90.2 (72.3; 96.8) |
| 5 years | 89.2% (71.9; 96.1) | 81.2% (52.1; 93.5) |

## Table S4. Recipient and graft characteristics of the analyzed cohort, stratified by living donation and brain-dead donor KT.

|  | **Total (n=99)** | **Living donor (n=51)** | **DBD**  **(n=48)** | **p-value** |
| --- | --- | --- | --- | --- |
| donor age [yr] | 69 (65; 84) | 68 (65; 79) | 69.5 (65; 84) | 0.130 |
| recipient age [yr] | 54 (13; 64) | 44 (20; 64) | 58.5 (13; 64) | **<0.001** |
| age gap donor-recipient | 20 (1; 63) | 25 (1; 55) | 11 (1; 63) | **<0.001** |
| proportion old for young KT | 56 (54.4%) | 32 (62.7%) | 24 (50%) | 0.141 |
| male recipient | 60 (60.6%) | 31 (60.8%) | 29 (60.4%) | 0.567 |
| BMI [kg/m²] | 25.2 (17.1; 69) | 24 (17.5; 35.8) | 26.7 (17.1; 69) | **0.004** |
| underlying disease  chronic GN  IgA nephropathy  ADPKD  other | 18 (18.2%)  12 (12.1%)  10 (10.1%)  15 (15.2%) | 11 (21.6%)  9 (17.6%)  3 (5.9%)  13 (25.5%) | 7 (14.6%)  3 (6.3%)  7 (14.6%)  2 (4.2%) | 0.191  0.262  0.075  0.135  **0.003** |
| hypertension | 84 (84.8%) | 44 (86.3%) | 40 (83.3%) | 0.449 |
| CAD | 14 (14.1%) | 3 (5.9%) | 11 (22.9%) | **0.016** |
| diabetes | 8 (8.1%) | 2 (3.9%) | 6 (12.5%) | 0.116 |
| history of smoking | 17 (17.2%) | 8 (15.7%) | 9 (18.8%) | 0.445 |
| pre-emptive KT | 14 (14.1%) | 14 (27.5%) | - | **<0.001** |
| ≥ 1 prior KT | 9 (9.1%) | 6 (11.8%) | 3 (6.3%) | 0.275 |
| waiting time [months] | 51.4 (0.23; 255) | 14.3 (0.2; 255.5) | 74.4 (10.5; 160.2) | **<0.001** |
| type of dialysis  haemodialysis  CAPD  peritoneal dialysis | 69 (69.7%)  4 (4%)  12 (12.1%) | 29 (56.9%)  2 (3.9%)  6 (11.8%) | 40 (83.3%)  2 (4.2%)  6 (12.5%) | 0.579 |
| HLA-MM | 3 (0; 6) | 3 (0; 6) | 3 (0; 6) | 0.061 |
| presence of PRA | 17 (17.2%) | 9 (17.6%) | 8 (16.7%) | 0.574 |

## Table S5. Intra- and postoperative results of the analyzed cohort, stratified by living donation vs. brain-dead donor KT.

|  | **Total (n=99)** | **Living donor**  **(n=51)** | **DBD**  **(n=48)** | **p-value** |
| --- | --- | --- | --- | --- |
| left-sided implantation | 36 (36.4%) | 17 (33.3%) | 19 (39.6%) | 0.416 |
| CIT [min] | 233 (4; 1783) | 152.5 (4; 998) | 906 (198; 1783) | **<0.001** |
| operating time [min] | 171 (84; 472) | 154 (84; 342) | 189 (111; 472) | **<0.001** |
| WIT [min] | 38.5 (17; 105) | 37 (21; 105) | 44 (17; 98) | 0.093 |
| intraoperative complications | 9 (9.1%) | 6 (11.8%) | 3 (6.3%) | 0.275 |
| postoperative complications classified as Clavien Dindo | 40 (40.4%) | 19 (37.3%) | 21 (43.8%) | 0.384 |
| grade 1 | 6 (6.1%) | 6 (11.8%) | - | **0.016** |
| grade 2 | 24 (24.2%) | 14 (27.5%) | 10 (20.8%) | 0.298 |
| grade 3a | 5 (5.1%) | - | 5 (10.4%) | **0.024** |
| grade 3b | 5 (5.1%) | 1 (2%) | 4 (8.3%) | 0.163 |
| ICU | 35 (35.4%) | 23 (45.1%) | 12 (25%) | **0.035** |
| length ICU stay [d] | 5 (0; 29) | 6 (0; 15) | 1.5 (0; 29) | 0.01 |
| DGF | 36 (36.4%) | 10 (19.6%) | 26 (54.2%) | **<0.001** |

## Table S6. Subgroup analysis comprising DBD or living kidney donations only, analyzing one, three- and five-years death censored graft survival and overall survival, compared between “old for young” vs. “very old for young” KT.

|  | **DBD only** | | **Living kidney donation only** | |
| --- | --- | --- | --- | --- |
|  | **“Old for young”** | **“Very old for young”** | **“Old for young”** | **“Very old for young”** |
| Graft survival |  |  |  |  |
| 1 year | 91.1% | 90.5% | 100% | 94% |
| 3 years | 86% | 90.5% | 100% | 94% |
| 5 years | 78.9% | 90.5% | 100% | 84.6% |
| Patient survival |  |  |  |  |
| 1 year | 100% | 95.2% | 100% | 100% |
| 3 years | 90.2% | 82.5% | 96.6% | 100% |
| 5 years | 90.2% | 82.5% | 96.6% | 100% |

## Table S7. Uni- and multivariate Cox Regression analysis to assess potential influencing factors on death-censored graft survival including 95% confidence intervals.

|  | **Univariate** | | **Multivariate** | |
| --- | --- | --- | --- | --- |
|  | HR (95%CI) | p-value | HR (95%CI) | p-value |
| type of kidney donation (ref.: living) | 2.47 (0.76; 8.03) | 0.132 | - | - |
| old for young (ref.) vs. very old for young KT | 1.75 (0.56; 5.46) | 0.338 | - | - |
| donor age | 1.05 (0.91; 1.22) | 0.486 | - | - |
| recipient age | 1.0 (0.95; 1.05) | 0.918 | - | - |
| age gap donor-recipient | 1.01 (0.96; 1.06) | 0.756 | - | - |
| BMI | 0.94 (0.82; 1.08) | 0.397 | - | - |
| hypertension | 1.93 (0.25; 14.99) | 0.531 | - | - |
| coronary artery disease | 5.47 (1.71; 17.46) | **0.004** |  | 0.069 |
| diabetes | 1.28 (0.16; 9.96) | 0.814 | - | - |
| history of smoking | 1.36 (0.3; 6.27) | 0.694 | - | - |
| pre-emptive KT | 0.42 (0.06; 3.3) | 0.412 | - | - |
| ≥ 1 prior KT | 1.57 (0.34; 7.2) | 0.563 | - | - |
| waiting time [years] | 1.16 (1.03; 1.31) | **0.018** |  | 0.106 |
| type of dialysis (ref. haemodialysis) | 0.47 (0.06; 3.7) | 0.475 | - | - |
| HLA-MM | 1.05 (0.71; 1.55) | 0.796 | - | - |
| presence of PRA | 5.54 (1.69; 18.18) | **0.005** | 8.32 (2.34; 29.53) | **0.001** |

## Table S8. Uni- and multivariate Cox Regression analysis to assess potential influencing factors on patient survival including 95% confidence intervals.

|  | **Univariate** | | **Multivariate** | |
| --- | --- | --- | --- | --- |
|  | HR (95%CI) | p-value | HR (95%CI) | p-value |
| type of kidney donation (ref.: living) | 4.74 (0.93; 24.09) | 0.060 | - | - |
| old for young (ref.) vs. very old for young KT | 1.78 (0.44; 7.07) | 0.421 | - | - |
| donor age | 1.11 (0.94; 1.32) | 0.214 | - | - |
| recipient age | 1.08 (0.99; 1.18) | 0.092 | - | - |
| age gap donor-recipient | 0.95 (0.89; 1.03) | 0.197 | - | - |
| BMI | 0.79 (0.63; 0.98) | **0.029** | 0.73 (0.58; 0.92) | **0.008** |
| hypertension | 1.23 (0.15; 10.13) | 0.845 | - | - |
| coronary artery disease | 11.67 (2.78; 48.84) | **<0.001** | 19.74 (3.3; 117.96) | **<0.001** |
| diabetes | 4.34 (0.87; 21.64) | 0.074 | - | - |
| history of smoking | 1.99 (0.4; 9.86) | 0.401 | - | - |
| pre-emptive KT | 0.04 (0.0; 107.2) | 0.417 | - | - |
| ≥ 1 prior KT | 2.78 (0.56; 13.86) | 0.213 | - | - |
| waiting time [years] | 1.17 (1.02; 1.33) | **0.027** | 1.28 (1.0; 1.64) | 0.054 |
| type of dialysis (ref. haemodialysis) | 0.04 (0.0; 88.5) | 0.405 |  |  |
| HLA-MM | 0.913 (0.58; 1.45) | 0.669 | - | - |
| presence of PRA | 4.44 (1.11; 17.76) | **0.035** |  | 0.106 |

## Fig. S1


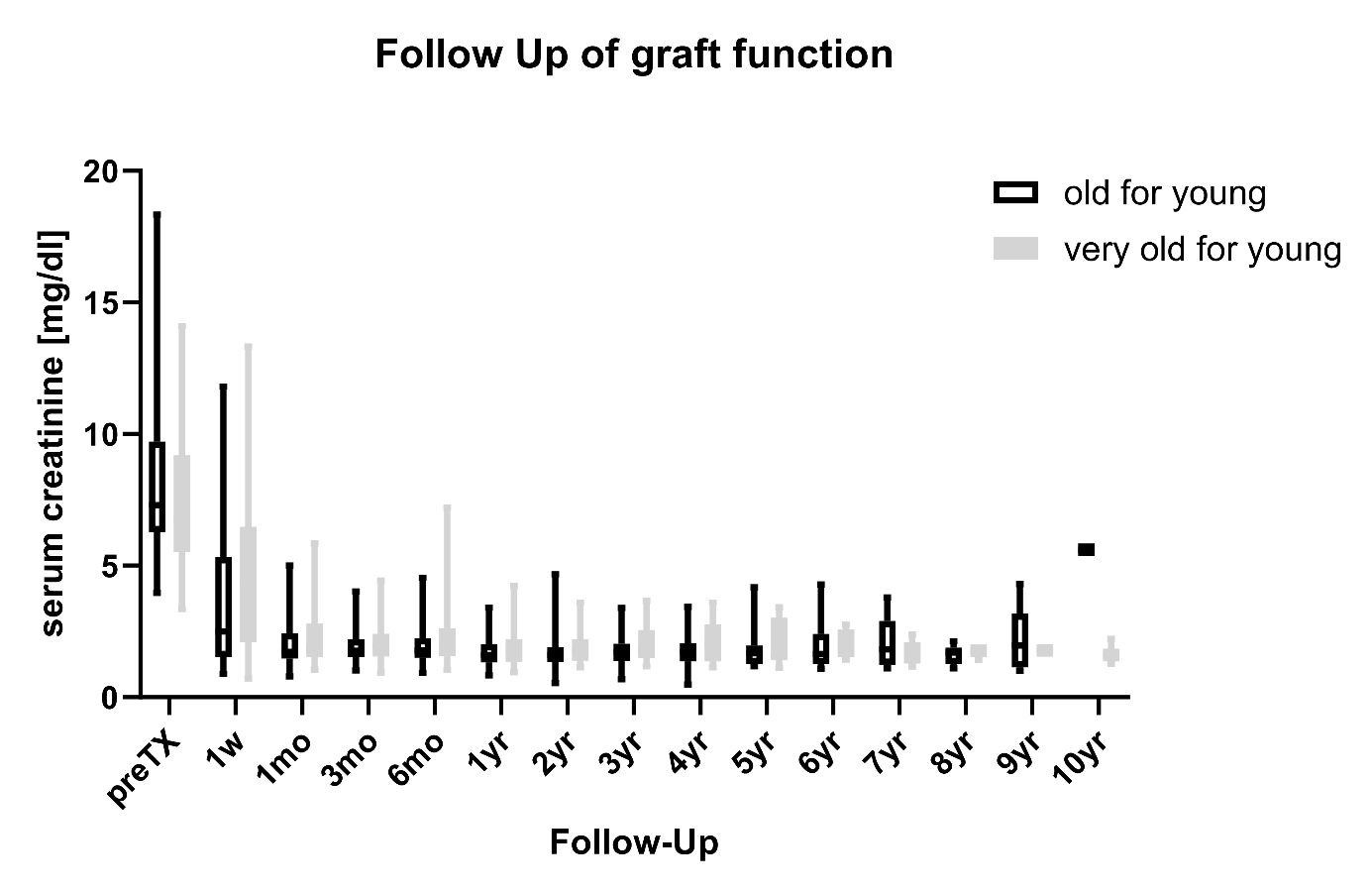


**Figure S1**: Boxplots indicating the follow-up of the graft function, compared between “old for young” vs. “very old for young” kidney transplantation.

## Fig. S2


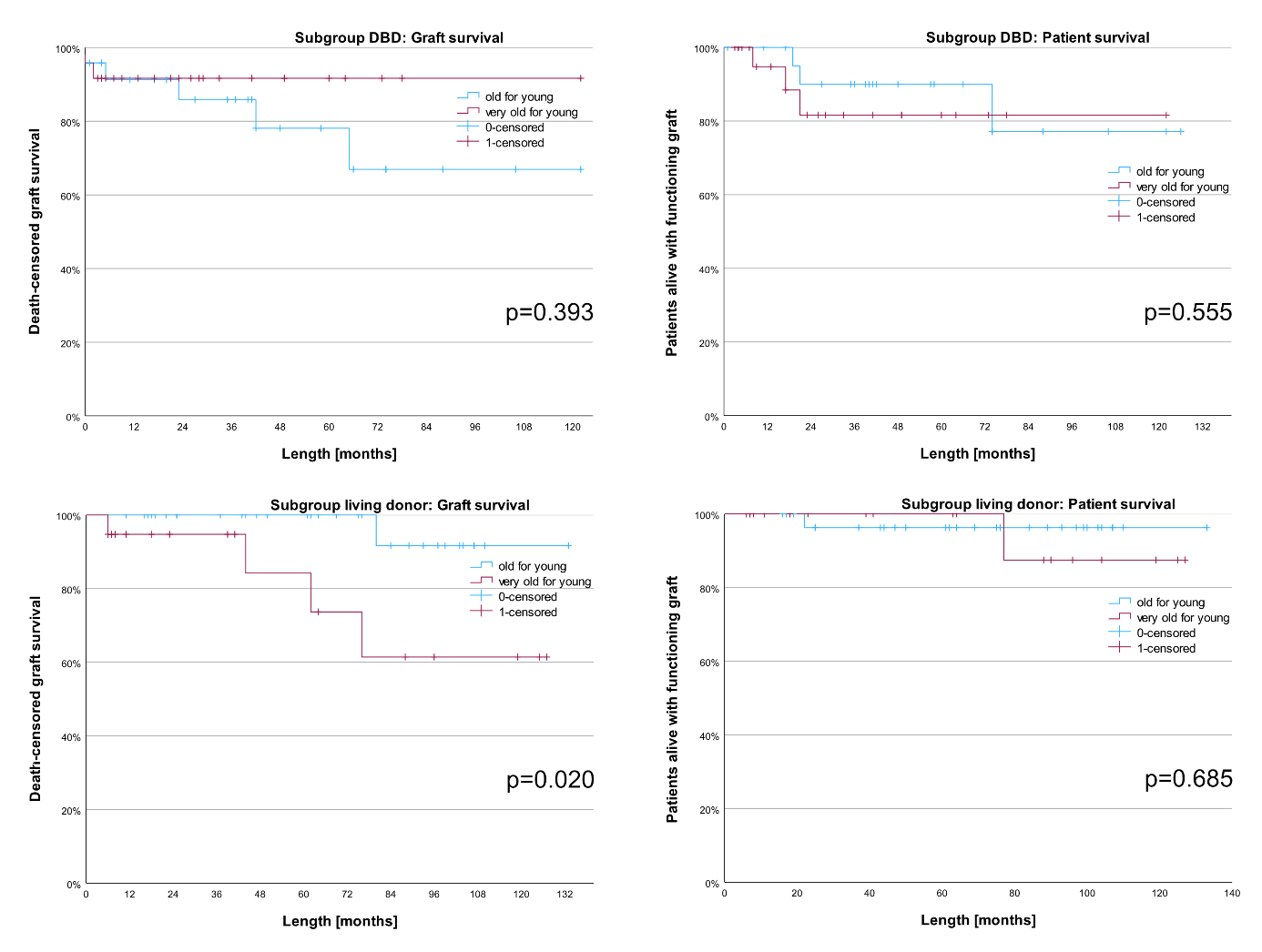


**Figure S2.** Death-censored graft (left column) and patient survival (right column), compared between “old for young” vs. “very old for young” kidney transplantation, but including DBD transplantations (upper row) or living kidney donations (lower row) only.
